# Deciphering the gene expression profile of peroxisome proliferator-activated receptor signaling pathway in the left atria of patients with mitral regurgitation

**DOI:** 10.1186/s12967-016-0871-3

**Published:** 2016-06-02

**Authors:** Mien-Cheng Chen, Jen-Ping Chang, Yu-Sheng Lin, Kuo-Li Pan, Wan-Chun Ho, Wen-Hao Liu, Tzu-Hao Chang, Yao-Kuang Huang, Chih-Yuan Fang, Chien-Jen Chen

**Affiliations:** Division of Cardiology, Department of Internal Medicine, Kaohsiung Chang Gung Memorial Hospital, Chang Gung University College of Medicine, 123 Ta Pei Road, Niao Sung District, Kaohsiung, 83301 Taiwan; Division of Cardiovascular Surgery, Kaohsiung Chang Gung Memorial Hospital, Kaohsiung, Taiwan; Division of Cardiology, Chang Gung Memorial Hospital, Chiayi, Taiwan; Graduate Institute of Biomedical Informatics, Taipei Medical University, Taipei, Taiwan; Department of Thoracic and Cardiovascular Surgery, Chang Gung Memorial Hospital, Chiayi, Taiwan

**Keywords:** Atrium, Genes, Mitral regurgitation

## Abstract

**Background:**

Differentially expressed genes in the left atria of mitral regurgitation (MR) pigs have been linked to peroxisome proliferator-activated receptor (PPAR) signaling pathway in the KEGG pathway. However, specific genes of the PPAR signaling pathway in the left atria of MR patients have never been explored.

**Methods:**

This study enrolled 15 MR patients with heart failure, 7 patients with aortic valve disease and heart failure, and 6 normal controls. We used PCR assay (84 genes) for PPAR pathway and quantitative RT-PCR to study specific genes of the PPAR pathway in the left atria.

**Results:**

Gene expression profiling analysis through PCR assay identified 23 genes to be differentially expressed in the left atria of MR patients compared to normal controls. The expressions of APOA1, ACADM, FABP3, ETFDH, ECH1, CPT1B, CPT2, SLC27A6, ACAA2, SMARCD3, SORBS1, EHHADH, SLC27A1, PPARGC1B, PPARA and CPT1A were significantly up-regulated, whereas the expression of PLTP was significantly down-regulated in the MR patients compared to normal controls. The expressions of HMGCS2, ACADM, FABP3, MLYCD, ECH1, ACAA2, EHHADH, CPT1A and PLTP were significantly up-regulated in the MR patients compared to patients with aortic valve disease. Notably, only ACADM, FABP3, ECH1, ACAA2, EHHADH, CPT1A and PLTP of the PPAR pathway were significantly differentially expressed in the MR patients compared to patients with aortic valve disease and normal controls.

**Conclusions:**

Differentially expressed genes of the PPAR pathway have been identified in the left atria of MR patients compared with patients with aortic valve disease and normal controls.

## Background

Mitral regurgitation (MR) is an important cause of heart failure secondary to valvular heart disease [1]. Structural remodeling associated with atrial enlargement developed in the left atrial myocardium of MR patients with heart failure [2–4]. Differential gene expression related to the left atrial structural remodeling of MR has been reported in the pig MR model [5]. Interestingly, gene ontology and pathway enrichment analysis of the differentially expressed genes in that study showed that peroxisome proliferator-activated receptor (PPAR) signaling pathway was identified in the KEGG pathway. However, specific genes of the PPAR signaling pathway that were differentially expressed in the left atrial myocardium of MR patients have never been explored.

The PPARs are ligand activated transcription factors that regulate genes important in cell differentiation, various metabolic processes, especially lipid and glucose homeostasis, insulin sensitivity, control of inflammatory processes and vascular integrity [6]. The family of PPARs comprises three isoforms: PPARα, PPARβ/δ and PPARγ [7]. PPARα is expressed mainly in metabolically active tissues, such as heart, liver, and skeletal muscle [6], and plays important physiologic roles in fatty acid oxidation and fatty acid metabolism.

In this study, we aim to explore the key element genes of the PPAR signaling pathway that were differentially expressed in the left atrial myocardium of MR patients vs. normal controls. The left atrial myocardium of patients with severe aortic valve disease was also used as a reference for gene analysis. The results from this study may identify specific genes of the PPAR signaling pathway that might be responsible for the atrial structural remodeling associated with atrial enlargement and progression of heart failure in patients with MR.

## Methods

### Patient population

This study enrolled 15 severe non-ischemic MR patients with heart failure and sinus rhythm (age 57 ± 9 years) and 7 patients with severe degenerative aortic valve disease and heart failure in sinus rhythm (age 60 ± 11 years; aortic stenosis in 1, aortic regurgitation in 4, combined aortic stenoregurgitation in 2). Exclusion factors include previous myocardial infarction, febrile disorder, infectious or inflammatory disease, autoimmune disease, malignancy, acute or chronic viral hepatitis or use of immunosuppressive drugs. Informed consent was obtained from each study patient, and the study protocol conforms to the ethical guidelines of the 1975 Declaration of Helsinki as reflected in a priori approval by the institution’s human research committee (102-2219C).

### Specimen storage

Atrial tissues of MR patients and aortic valve disease patients were sampled from the left atrial free wall during surgery. After excision, some atrial tissues were immediately frozen in liquid nitrogen. Additionally, some atrial tissues were placed into a Tissue Tek^®^ container which was then filled with Tissue Tek^®^ optimum cutting temperature compound (Sakura^**®**^ Finetek, CA, USA) and these samples were frozen in liquid nitrogen for later histochemical study.

### PCR assay and data processing

RNAs were extracted from the myocardial tissue using a RiboPureTM kit (Ambion, Grand Island, NY, USA) according to the manufacturer’s protocol. RNA quality was assessed using an Agilent 2100 Bioanalyzer (Agilent Technologies Inc, Santa Clara, CA, USA). PPAR pathway related resources were obtained using information from website (https://www.qiagen.com/tw/shop/genes-and-pathways/pathway-details/?pwid=367). A total of 84 genes of the PPAR signaling pathway were examined by RT^2^ profiler PCR array (Qiagen, CA, USA) according to the manufacturer’s directions. Ribosomal protein, large, P0 (RPLP0) gene served as the endogenous control. Fold-change values greater than one indicated a positive- or an up-regulation, and fold-change values less than one indicated a negative or down-regulation.

### Quantitative determination of RNAs by real-time RT-PCR

The RNA samples were quantified using a spectrophotometer. First-strand cDNAs were synthesized with reverse transcriptase and oligo (dT) primers. Real-time quantitative PCR was performed on the ABI Prism 7500 FAST sequence detection system (Applied Biosystems, CA, USA), using SYBR Green PCR Master Mix (Qiagen, CA, USA). The results were normalized against RPLP0 gene expression (the endogenous control). The selected genes (mRNAs) and primer sequences are presented in Table 1. The Primer3 Input (version 0.4.0) (http://bioinfo.ut.ee/primer3-0.4.0/) was used to design the primers. Quantitative RT-PCR values were presented in ΔCq units.

**Table 1:** Primer sequences for real-time PCR

Gene name	Forward primer	Reverse primer
ACAA2	TGC GTT TTG GAA CCA AGC	CAT GCT GAT CTG TTA ATG ATA CCC
ACADM	AGG AGC CAT TGA TGT GTG C	CTG CTT TGG TCT TTA TAC CAG CTA
APOA1	CCT TGG GAA AAC AGC TAA ACC	CCA GAA CTC CTG GGT CAC A
CPT1A	ACA ACA AAA GCC CCT GAC TG	AGG GCA GAG AGA GCT ACA TCC
CPT1B	GCT GAA GGT TGG AGA AAT GC	CCT CAT GCC TGT GAG CTG
CPT2	TGA CCG ACA CTT GTT TGC TC	GAG CTC AGG CAA GAT GAT CC
ECH1	GTA CTG TGC CCA GGA TGC TT	CTC TGG TTC CCG ATG ACC T
EHHADH	CCT GGG CTG TCA CTA TAG GAT T	AGA AGC TGG GTT CCT CTT GC
ETFDH	CCC GGG ATA AGG ACA AGA G	CAT CTG CTT CTT CTG CAA ACC
FABP3	CTG GGC ACC TGG AAG CTA	TGG TAG CAA AAC CCA CAC C
PPARA	CCG CAA TGG ACC ATG TAA C	CAG CTC TAG CAT GGC CTT TT
HMGCS2	GCG TCC CGT CTA AAG GTG T	ACC AGC TAA GAG TGG GAT CTT AAA
KLF10	AGC CAA CCA TGC TCA ACT TC	CTC TTT TGG CCT TTC AGA AAT C
MMP9	GAA CCA ATC TCA CCG ACA GG	GCC ACC CGA GTG TAA CCA TA
PPARGC1B	TGT TTC ATC AGT ATG CTT TGC AC	CAA ATT TGG GCA GTT GGA TT
SLC27A1	TGC CGA GAG TGG AAC ACA C	AAA AGC AGC TGG ACC CTA CA
SLC27A6	GGG CTT TTG GTT GTA CTG CT	AAA TTT CTT CTT TAA CAC ACA AGT GG
SMARCD3	CTG CTC CTC ATG CTG GAC TA	GCC TGG ACA ATG GCT GAG
SORBS1	GAC GTC ATG ATG ATA AAG AGA TGA G	GAG GAA GCT CCT TTA GTG TCT GA
PLTP	CTT CGG GGG AAC CTT CAA	GTG GTA GAG GAC AGG GCA GA
MLYCD	TTG CAC GTG GCA CTG ACT	GGA TGT TCC TTC ACG ATT GC
RPLP0	GGC ACC ATT GAA ATC CTG AG	GAA GGG GGA GAT GTT GAG C

*ACAA2* acetyl-CoA acyltransferase 2, *ACADM* acyl-CoA dehydrogenase, C-4 to C-12 straight chain, *APOA1* Apolipoprotein A-I, CPT1A carnitine palmitoyltransferase 1A (liver), *CPT1B* carnitine palmitoyltransferase 1B (muscle), *CPT2* carnitine palmitoyltransferase 2, *ECH1* enoyl CoA hydratase 1, peroxisomal, *EHHADH* enoyl-CoA, hydratase/3-hydroxyacyl CoA dehydrogenase, *ETFDH* electron-transferring-flavoprotein dehydrogenase, *FABP3* fatty acid binding protein 3, muscle and heart (mammary-derived growth inhibitor), *PPARA* peroxisome proliferator-activated receptor alpha, *HMGCS2* 3-hydroxy-3-methylglutaryl-CoA synthase 2 (mitochondrial), *KLF10* Kruppel-like factor 10, MMP9 Matrix metallopeptidase 9, *PPARGC1B* peroxisome proliferator-activated receptor gamma, coactivator 1 beta, *SLC27A1* Solute carrier family 27 (fatty acid transporter), member 1, *SLC27A6* solute carrier family 27 (fatty acid transporter), member 6, *SMARCD3* SWI/SNF related, matrix associated, actin dependent regulator of chromatin, subfamily d, member 3, *SORBS1* sorbin and SH3 domain containing 1, *PLTP* phospholipid transfer protein, *MLYCD* malonyl-CoA decarboxylase, *RPLP0* ribosomal protein, large, P0

### Western blotting

Protein concentrations of atrial myocardial tissues were determined by the Bradford method (Bio-Rad) according to the supplier’s instructions. The HeLa cell lysate (Santa Cruz, Texas, USA) served as positive control. 20 μg protein extracts were electrophoresed on a 12 % acrylamide SDS-PAGE gel and immunoblotted onto PVDF membranes. The membranes were blocked for 1 h in PBST containing 5 % w/v nonfat dry milk. The primary antibodies, including anti-ACADM and ECH1 (Abcam, Cambridge, USA), were used to react with the blots at room temperature for 2 h. Immunoreactivity was revealed with horseradish peroxidase-conjugated secondary antibody. All specific values of proteins evaluated were standardized to GAPDH (GeneTex, CA, USA).

### Oil red O staining

Left atrial tissues were sliced into 8-µm sections, stained with Oil red O to visualize lipid accumulation (ScyTek Laboratories, Utah, USA) according to the manufacturer’s directions. Sections were mounted and visualized using an Olympus BX51 microscope. The Oil red O stained area per myocyte was analyzed by Cellsens Dimension (Olympus, JAPAN) with at least 100 randomly chosen myocytes per each sample.

### Statistical analysis

Data are presented as mean ± SD (baseline characteristics) or SEM (gene, proteins, and Oil red O staining expressions). Categorical variables were compared using Chi square test or Fisher exact test as appropriate. Continuous variables among 3 groups were analyzed by the Kruskal–Wallis Test, and continuous variables between 2 groups were analyzed by the Mann–Whitney Test. Statistical analysis was performed using commercial statistical software (IBM SPSS Statistics 22). A *P* value of <0.05 was considered statistically significant.

## Results

### Baseline characteristics of patients studied

Table 2 lists the clinical characteristics of the MR patients with heart failure and patients with aortic valve disease and heart failure. There was no significantly difference in heart failure status between MR patients with heart failure and patients with aortic valve disease and heart failure. The two groups did not significantly differ in age, prevalence of hypertension and diabetes mellitus, and use of β-blockers and calcium channel blockers.

**Table 2:** Baseline clinical characteristics of the study patients

	MR (n = 15)	AVD (n = 7)	NC (n = 3)	*P* value
Age (years)	57 ± 9	60 ± 11		0.458
Male (%)	6 (40.0 %)	6 (85.7 %)		0.059
Smoking (%)	2 (13.3 %)	1 (14.3 %)		0.705
Body mass index (kg/m^2^)	23.5 ± 2.3	24.2 ± 3.3		0.259
Hypertension (%)	7 (46.7 %)	4 (57.1 %)		0.500
Diabetes mellitus (%)	2 (13.3 %)	1 (14.3 %)		0.705
Hyperlipidemia (%)	6 (40.0 %)	2 (28.6 %)		0.490
NYHA				0.506
Functional class I (%)	2 (13.3 %)	1 (14.3 %)		
Functional class II (%)	7 (46.7 %)	3 (42.9 %)		
Functional class III (%)	6 (40.0 %)	2 (28.6 %)		
Functional class IV (%)	0 (0.0 %)	1 (14.3 %)		
Aortic valve disease (%)	0 (0.0 %)	7 (100.0 %)		<0.001
Tricuspid regurgitation (%)	7 (46.7 %)	1 (14.3 %)		0.161
β-blockers (%)	5 (33.3 %)	0 (0.0 %)		0.114
Calcium channel blockers (%)	6 (40.0 %)	3 (42.9 %)		0.628
Angiotensin converting enzyme inhibitors or angiotensin II receptor blockers (%)	12 (80.0 %)	3 (42.9 %)		0.107
Creatinine (mg/dl)	0.9 ± 0.7	1.0 ± 0.3		0.139
White blood cell count (10^3^/uL)	6.3 ± 1.5	5.6 ± 1.8		0.289
Left atrial diameter (mm)	45.5 ± 6.0	38.9 ± 5.8		0.028
Left atrial maximal volume (mL)	87.3 ± 42.6	42.5 ± 25.6		0.032
Left atrial ejection fraction (%)	49.7 ± 11.9	45.6 ± 18.7		0.654
Left ventricular end-diastolic diameter (mm)	58.2 ± 7.3	59.9 ± 12.7		0.397
Left ventricular ejection fraction (%)	67.3 ± 11.5	61.6 ± 12.9		0.340

Data are presented as mean ± SD or number (percentage)

*AVD* aortic valve disease, *MR* mitral regurgitation, *NC* purchased normal controls, *NYHA* New York Heart Association, P value MR vs. AVD

The left atrial size was significantly larger in the MR patients with heart failure than patients with aortic valve disease and heart failure (*P* < 0.05). The MR patients with heart failure and patients with aortic valve disease and heart failure did not significantly differ in left ventricular size and ejection fraction.

### Gene expression profiling analysis of the PPAR signaling pathway through PCR assay in the left atrium of MR patients with heart failure vs. normal controls

To determine the effect of MR and heart failure on the gene expression of PPAR signaling pathway, we compared the expression profile by PCR assay in the left atria of MR patients with heart failure (n = 5) to normal controls (n = 3; 76-year-old Caucasian female, 24-year-old Caucasian male and 27-year-old Caucasian male, purchased from BioChain, Newark, CA, USA). Differentially expressed genes were filtered using criteria of a fold-change in expression level more than 2 or less than 0.5 and *P* value <0.1 in the left atria of MR patients with heart failure compared to normal controls. A total of 23 differentially expressed genes of PPAR signaling pathway were identified to be differentially expressed in the left atrial tissues of MR patients with heart failure compared to normal controls (Table 3). Therefore, we focused on deciphering and experimental validation of these 23 genes in the following section in order to identify some of the differentially expressed genes of the PPAR signaling pathway that might be responsible for the structural remodeling of left atria in the MR patients [2–4].

**Table 3:** Selected signature mRNA expression of the PPAR signaling pathway through PCR assay in the left atria of mitral regurgitation patients with heart failure vs. normal control

Symbol	Description	Fold change	*P* value
Lipid metabolism			
APOA1	Apolipoprotein A-I	21.724	0.005449
HMGCS2	3-hydroxy-3-methylglutaryl-CoA synthase 2	6.5229	0.083
ACADM	Acyl-CoA dehydrogenase, C-4 to C-12 straight chain	3.8861	0.031596
FABP3	Fatty acid binding protein 3, muscle and heart (mammary-derived growth inhibitor)	3.496	0.027634
ETFDH	Electron-transferring-flavoprotein dehydrogenase	3.4251	0.001157
MLYCD	Malonyl-CoA decarboxylase	3.372	0.000504
ECH1	Enoyl CoA hydratase 1, peroxisomal	3.2082	0.006862
CPT1B	Carnitine palmitoyltransferase 1B	3.1846	0.0363
CPT2	Carnitine palmitoyltransferase 2	3.1829	0.071083
SLC27A6	Solute carrier family 27 (fatty acid transporter), member 6	3.0759	0.099593
ACAA2	Acetyl-CoA acyltransferase 2	2.9113	0.005252
EHHADH	Enoyl-CoA, hydratase/3-hydroxyacyl CoA dehydrogenase	2.8434	0.03971
SLC27A1	Solute carrier family 27 (fatty acid transporter), member 1	2.8389	0.003705
ACSL3	Acyl-CoA synthetase long-chain family member 3	2.2958	0.028469
CPT1A	Carnitine palmitoyltransferase 1A	1.9237	0.023573
PLTP	Phospholipid transfer protein	0.2311	0.027612
Coactivator			
SMARCD3	SWI/SNF related, matrix associated, actin dependent regulator of chromatin, subfamily d, member 3	2.9058	0.037134
PPARGC1B	Peroxisome proliferator-activated receptor gamma, coactivator 1 beta	2.6275	0.080693
Signaling and stimulation of insulin			
SORBS1	Sorbin and SH3 domain containing 1	2.8643	0.03188
Transcription factor			
PPARA	Peroxisome proliferator-activated receptor alpha	2.2651	0.026418
KLF10	Kruppel-like factor 10	2.093	0.095046
Mediating protein–protein interactions			
FGR	Gardner-Rasheed feline sarcoma viral (v-fgr) oncogene homolog	0.121	0.077129
Adipocyte differentiation			
MMP9	Matrix metallopeptidase 9	0.0737	0.081105

### Quantitative PCR validation of differentially expressed mRNAs of the PPAR signaling pathway in the left atria among MR patients with heart failure, patients with aortic valve disease and heart failure, and normal controls

The left atrial myocardium of patients with severe aortic valve disease and heart failure was also used as a reference for gene analysis of the PPAR signaling pathway.

The expressions of APOA1 (4.65 ± 0.52 vs. 7.37 ± 0.81, *P* = 0.011), ACADM (1.40 ± 0.09 vs. 3.38 ± 0.46, *P* = 0.001), FABP3 (−2.83 ± 0.19 vs. −1.58 ± 0.32, *P* = 0.006), ETFDH (2.41 ± 0.13 vs. 4.29 ± 0.21, *P* = 0.001), ECH1 (0.25 ± 0.10 vs. 2.18 ± 0.17, *P* = 0.001), CPT1B (3.65 ± 0.18 vs. 6.06 ± 0.22, *P* = 0.001), CPT2 (3.75 ± 0.16 vs. 6.22 ± 0.29, *P* = 0.001), SLC27A6 (3.29 ± 0.19 vs. 5.76 ± 0.71, *P* = 0.005), ACAA2 (2.63 ± 0.11 vs. 4.25 ± 0.34, *P* = 0.001), SMARCD3 (2.93 ± 0.11 vs. 4.33 ± 0.33, *P* = 0.002), SORBS1 (6.08 ± 0.16 vs. 7.71 ± 0.66, *P* = 0.005), EHHADH (4.65 ± 0.19 vs. 5.92 ± 0.43, *P* = 0.017), SLC27A1 (3.83 ± 0.16 vs. 5.94 ± 0.37, *P* = 0.001), PPARGC1B (4.61 ± 0.23 vs. 8.10 ± 0.71, *P* = 0.001), PPARA (4.84 ± 0.17 vs. 6.80 ± 0.37, *P* = 0.001) and CPT1A (5.60 ± 0.17 vs. 6.82 ± 0.33, *P* = 0.005) in the left atria were significantly up-regulated in the MR patients with heart failure (n = 14) compared to normal controls (n = 6; 24-year-old Caucasian male, 27-year-old Caucasian male, 30-year-old Asian male, 60-year-old Caucasian female, 76-year-old Caucasian female and 77-year-old Caucasian male, purchased from BioChain, Newark, CA, USA). Whereas the expression of PLTP (4.22 ± 0.14 vs. 2.77 ± 0.48, *P* = 0.006) in the left atria was significantly down-regulated in the MR patients with heart failure compared to normal controls.

The expressions of ETFDH (3.12 ± 0.36 vs. 4.29 ± 0.21, *P* = 0.037), ECH1 (1.10 ± 0.24 vs. 2.18 ± 0.17, *P* = 0.010), CPT1B (3.76 ± 0.31 vs. 6.06 ± 0.22, *P* = 0.004), CPT2 (3.81 ± 0.19 vs. 6.22 ± 0.29, *P* = 0.004), SMARCD3 (2.84 ± 0.14 vs. 4.33 ± 0.33, *P* = 0.006), SORBS1 (6.08 ± 0.33 vs. 7.71 ± 0.66, *P* = 0.037), SLC27A1 (3.57 ± 0.27 vs. 5.94 ± 0.37, *P* = 0.004), PPARGC1B (4.34 ± 0.24 vs. 8.10 ± 0.71, *P* = 0.004) and PPARA (5.10 ± 0.31 vs. 6.80 ± 0.37, *P* = 0.010) in the left atria were significantly up-regulated in the patients with aortic valve disease and heart failure (n = 7) compared to normal controls (n = 6). Whereas the expression of PLTP (5.08 ± 0.15 vs. 2.77 ± 0.48, *P* = 0.006) in the left atria was significantly down-regulated in the patients with aortic valve disease and heart failure compared to normal controls.

The expressions of HMGCS2 (10.18 ± 0.52 vs. 12.35 ± 0.23, *P* = 0.011), ACADM (1.40 ± 0.09 vs. 2.18 ± 0.36, *P* = 0.039), FABP3 (−2.83 ± 0.19 vs. −1.92 ± 0.24, *P* = 0.011), MLYCD (11.96 ± 0.20 vs. 13.03 ± 0.20, *P* = 0.006), ECH1 (0.25 ± 0.10 vs. 1.10 ± 0.24, *P* = 0.008), ACAA2 (2.63 ± 0.11 vs. 3.64 ± 0.22, *P* = 0.002), EHHADH (4.65 ± 0.19 vs. 5.49 ± 0.22, *P* = 0.014), CPT1A (5.60 ± 0.17 vs. 6.28 ± 0.13, *P* = 0.017) and PLTP (4.22 ± 0.14 vs. 5.08 ± 0.15, *P* = 0.004) in the left atria were significantly up-regulated in the MR patients with heart failure compared to patients with aortic valve disease and heart failure.

**Fig. 1:**
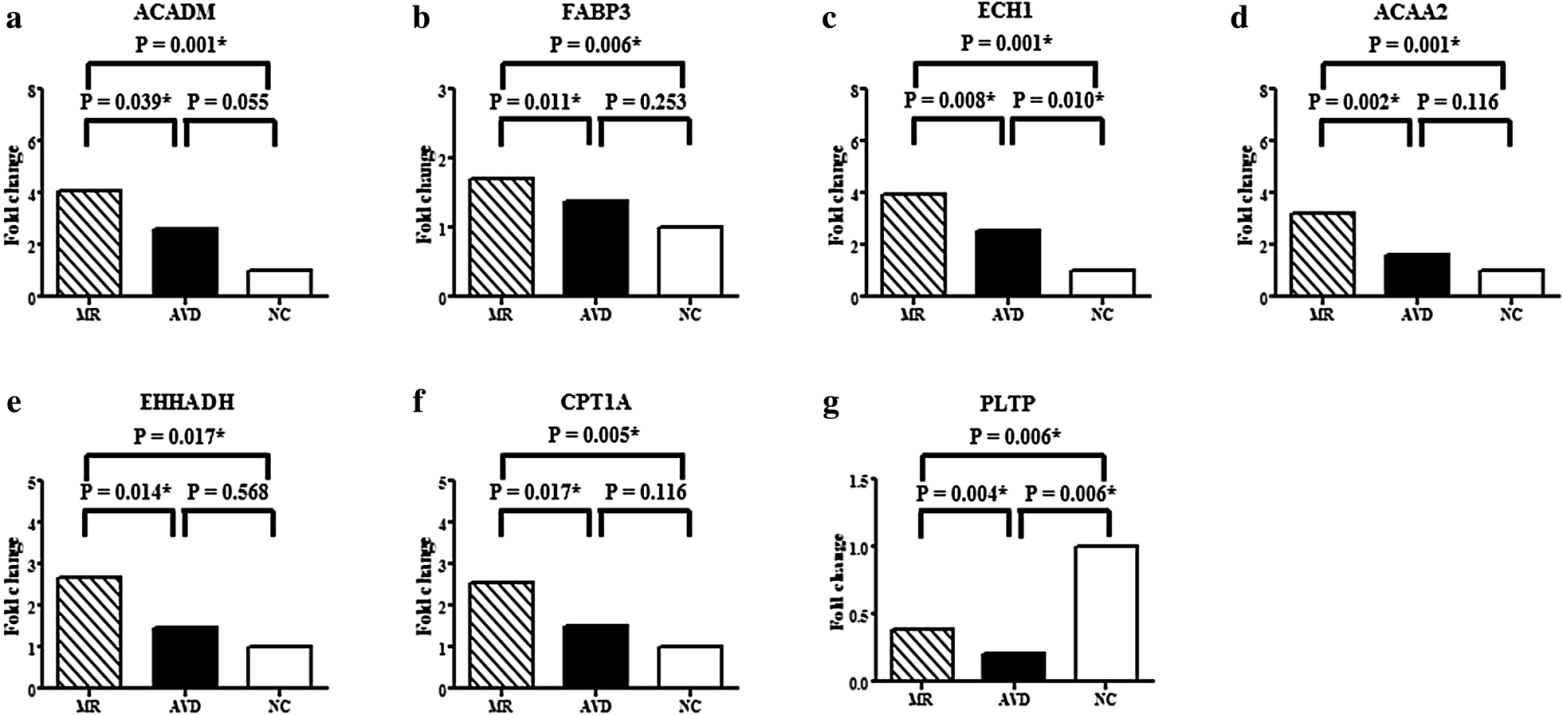
Quantitative determination of mRNAs of (**a**) Acyl-CoA dehydrogenase, C-4 to C-12 straight chain (ACADM), **b** Fatty acid binding protein 3 (FABP3), **c** Enoyl CoA hydratase 1 (ECH1), **d** Acetyl-CoA acyltransferase 2 (ACAA2), **e** Enoyl-CoA, hydratase/3-hydroxyacyl CoA dehydrogenase (EHHADH), **f** Carnitine palmitoyltransferase 1A (CPT1A), **g** Phospholipid transfer protein (PLTP) by real-time RT-PCR in the left atria of mitral regurgitation patients with heart failure (MR), patients with aortic valve disease and heart failure (AVD), and purchased normal controls (NC). *P < 0.05

Notably, only ACADM, FABP3, ECH1, ACAA2, EHHADH, CPT1A and PLTP of the PPAR signaling pathway were differentially expressed in the left atria of MR patients compared to patients with aortic valve disease and normal controls. The expressions of ACADM, FABP3, ECH1, ACAA2, EHHADH and CPT1A in the left atria were significantly up-regulated in the MR patients with heart failure compared to patients with aortic valve disease and heart failure and normal controls (Fig. 1). However, MR patients with heart failure had significantly up-regulated PLTP expression in the left atria compared to patients with aortic valve disease and heart failure but had significantly down-regulated PLTP expression in the left atria compared to normal controls (Fig. 1).

### The expression of fatty acid oxidation enzymes (ACADM, ECH1) in the left atria among MR patients with heart failure, patients with aortic valve disease and heart failure, and normal controls

**Fig. 2:**
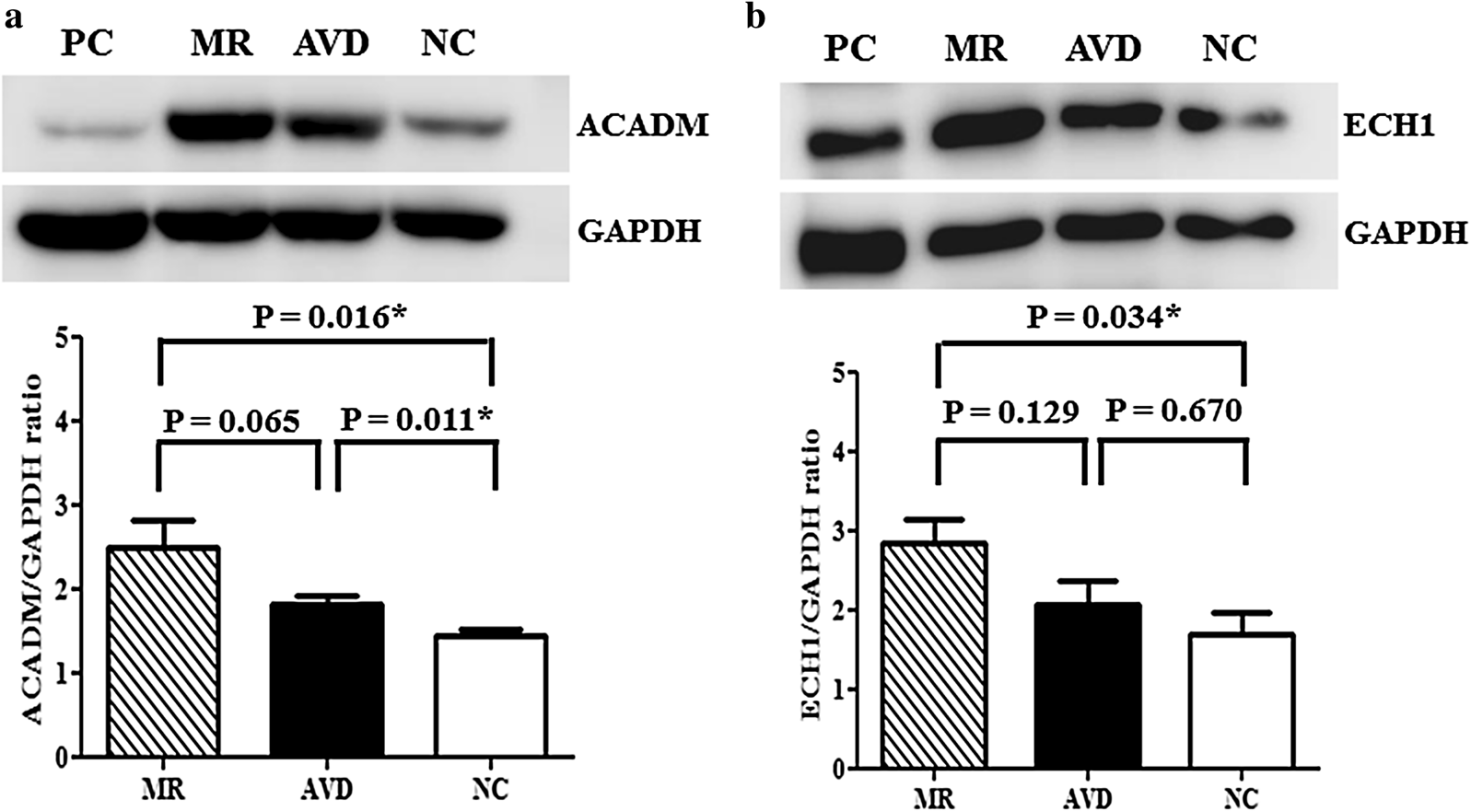
The expressions of (**a**) Acyl-CoA dehydrogenase, C-4 to C-12 straight chain (ACADM) and **b** Enoyl CoA hydratase 1 (ECH1) in the tissue extracts were determined by Western blotting in the left atria of mitral regurgitation patients with heart failure (MR) (n = 10), patients with aortic valve disease and heart failure (AVD) (n = 6), and purchased normal controls (NC) (n = 4). *P < 0.05. HeLa cell lysate served as positive control (PC)

The expressions of ACADM (2.49 ± 0.34 vs. 1.43 ± 0.09, *P* = 0.016) and ECH1 (2.84 ± 0.30 vs. 1.70 ± 0.27, *P* = 0.034) proteins in the left atria were significantly up-regulated in the MR patients with heart failure (n = 10) compared to normal controls (n = 4; 49-year-old African American male, 60-year-old Caucasian female and 62-year-old Caucasian female, purchased from BioChain, Newark, CA, USA and 35-year-old Asian female, purchased from G-bioscience, St Louis, MO, USA) (Fig. 2).

The expression of ACADM protein (1.81 ± 0.11 vs. 1.43 ± 0.09, *P* = 0.011) in the left atria was significantly up-regulated in the patients with aortic valve disease and heart failure (n = 6) compared to normal controls (n = 4) (Fig. 2). The expression of ECH1 protein (2.07 ± 0.30 vs. 1.70 ± 0.27, *P* = 0.670) in the left atria was up-regulated in the patients with aortic valve disease and heart failure (n = 6) compared to normal controls (n = 4), although the difference did not reach statistical significance.

The expressions of ACADM (2.49 ± 0.34 vs. 1.81 ± 0.11, *P* = 0.065) and ECH1 (2.84 ± 0.30 vs. 2.07 ± 0.30, *P* = 0.129) proteins in the left atria were up-regulated in the MR patients with heart failure (n = 10) compared to patients with aortic valve disease and heart failure (n = 6), although the difference did not reach statistical significance (Fig. 2).

### Left atrial myocytes fat staining

**Fig. 3:**
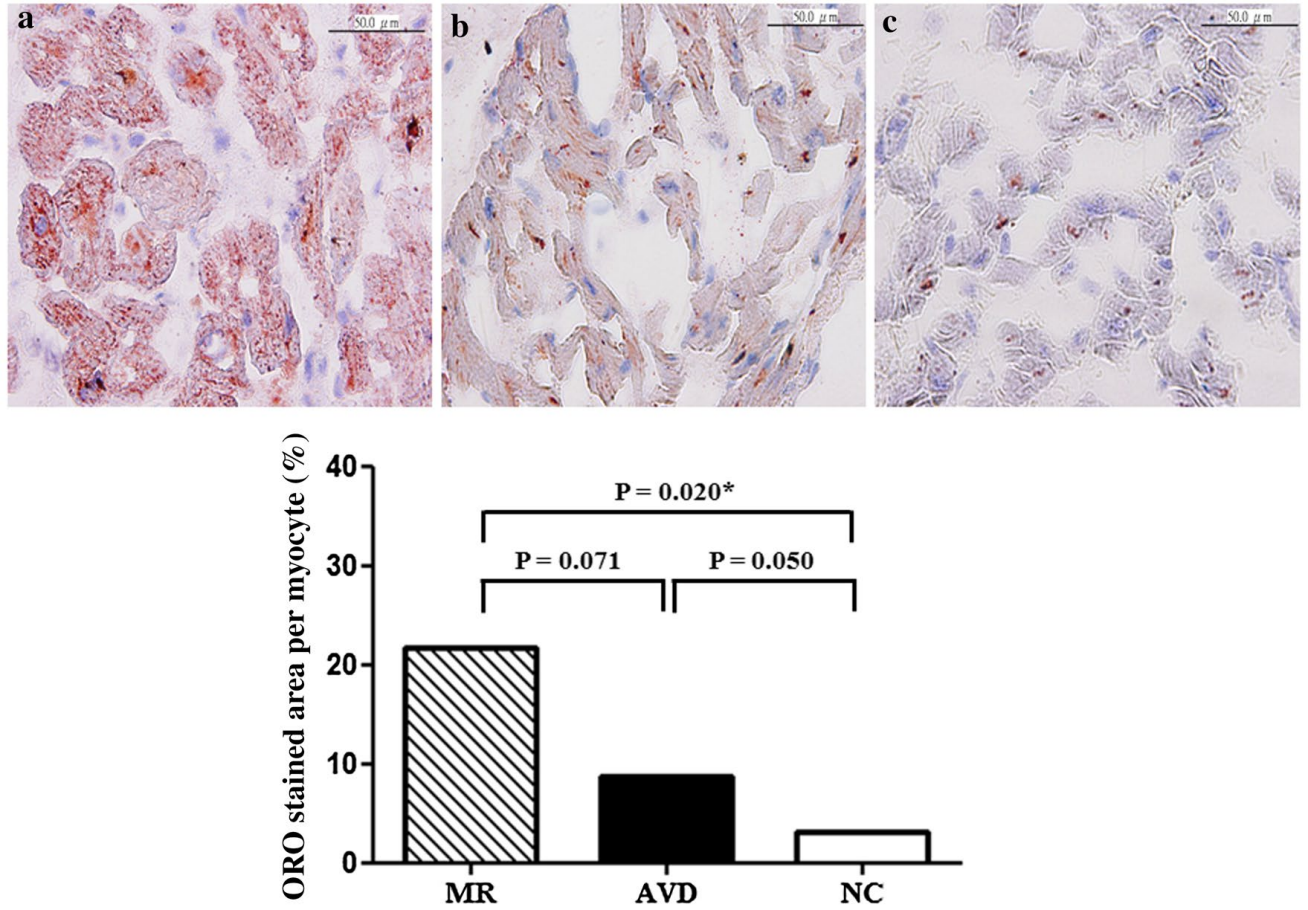
Lipid deposition with Oil red O (ORO) staining in left atrial myocytes of (**a**) mitral regurgitation patients with heart failure (MR) (n = 6), **b** patients with aortic valve disease and heart failure (AVD) (n = 3), and **c** purchased normal controls (NC) (n = 3). Percentage of area stained with ORO per myocyte in all groups. *P < 0.05

The percentage of area stained with Oil red O (lipid droplets) per myocyte in the left atria was significantly higher in the MR patients with heart failure (n = 6) compared to normal controls (n = 3; 49-year-old African American male, 76-year-old Caucasian female, and 77-year-old Caucasian male, purchased from BioChain, Newark, CA, USA) (21.76 ± 3.87 vs. 3.09 ± 0.70 %, *P* = 0.020) (Fig. 3). The percentage of area stained with Oil red O (lipid droplets) per myocyte in the left atria was higher in the MR patients with heart failure (n = 6) compared to patients with aortic valve disease and heart failure (n = 3) (21.76 ± 3.87 vs. 8.75 ± 0.70 %, *P* = 0.071). The percentage of area stained with Oil red O (lipid droplets) per myocyte in the left atria was higher in the patients with aortic valve disease and heart failure (n = 3) compared to normal controls (n = 3) (8.75 ± 0.70 vs. 3.09 ± 0.70 %, *P* = 0.050).

## Discussion

This study identifies and reports the altered gene expression pattern of the PPAR signaling pathway in the left atria among MR patients with heart failure, patients with aortic valve disease and heart failure, and normal controls without valve disease and heart failure. Notably, seven genes (ACADM, FABP3, ECH1, ACAA2, EHHADH, CPT1A and PLTP) of the PPAR signaling pathway were differentially expressed in the left atria of MR patients compared to patients with aortic valve disease and normal controls.

The PPAR transcriptional regulatory complex controls the expression of fatty acid utilization genes by binding to specific promoter DNA response elements with its heterodimeric partner, the retinoid X receptor, and interacting with PPARγ coactivator-1α to recruit other cofactors with histone acetylase activity to initiate gene transcription for fatty acid oxidation [8]. The myocardium utilizes primarily fatty acids for ATP production via mitochondrial fatty acid oxidation. However, altered expression of the fatty acid oxidation enzymes can impair mitochondrial metabolism and lead to pathologic remodeling of myocardium, probably through lipotoxicity, reactive oxidative stress overproduction, and ATP deficiency [9–11]. Our prior studies showed that altered mitochondrial function and reactive oxidative stress overproduction due to nox2 containing NADPH oxidase activity developed in the atria of MR patients with heart failure [12, 13]. Moreover, heart-specific overexpression of PPAR induced several target genes involved in fatty acid utilization and increased cardiac fatty acid uptake and oxidation [8]. In contrast, heart-specific overexpression of PPAR markedly diminished the expression of genes involved in glucose metabolism leading to impaired glucose uptake and utilization [8]. Of note, our prior study showed that glycogen accumulation increased in the atrial myocytes of MR patients [4]. Moreover, this study showed that lipid accumulation in the atrial myocytes was significantly increased in the MR patients with heart failure compared to normal controls.

Acyl-CoA dehydrogenase, C-4 to C-12 straight chain (ACADM) gene provides instructions for making an enzyme called medium-chain acyl-CoA dehydrogenase, which functions within mitochondria and is essential for fatty acid oxidation to metabolize medium-chain fatty acids [14].

Fatty-acid-binding protein 3, muscle and heart (FABP3), also known as heart-type FABP, is an intracellular lipid-binding protein for transporting fatty acids and other lipophilic substances from the cytoplasm to the nucleus and subsequently, PPAR activation by fatty acid ligands [8, 15]. Glatz JF et al. [16] reported that FABP overexpression in skeletal muscle increased fatty acids transported into the muscle cell and consequently, fatty acid oxidation was increased. On the other hand, PPAR can regulate the expression of FABP3 [8].

Enoyl-CoA hydratase1 (ECH1), a mitochondrial β-oxidation enzyme, has been shown to play an important role for mitochondrial integrity and function [17]. The expression of ECH1 in tissue is associated with nitric oxide availability [18] and a decreased production of nitric oxide by the mitochondrial form of nitric oxide synthase has been proposed as a cause of decreased mitochondrial biogenesis, resulting in impairment of cellular turnover, tissue regeneration and lipid metabolism [19].

Acetyl-CoA acyltransferase 2 (ACAA2) encoding protein catalyzes the last step of the mitochondrial fatty acid β-oxidation spiral. Additionally, ACAA2 has been demonstrated to have antiapoptotic effects, which provided a possible linkage between fatty acid metabolism and apoptosis of cells [20].

Enoyl-CoA, hydratase/3-hydroxyacyl CoA dehydrogenase (EHHADH), regulated and mediated by PPARα, encodes a protein that is an l-bifunctional enzyme essential for the peroxisomal β-oxidation pathway to the breakdown of very long chain fatty acids and is indispensable for the production of medium-chain dicarboxylic acids [21].

Carnitine palmitoyltransferase 1 (CPT1) is one of the carnitine cycle enzymes that plays a role in the transportation of long-fatty acids into the mitochondria for ß-oxidation that allows the body to process fats to provide energy during times of fasting and illness [22].

Phospholipid transfer protein (PLTP) is a widely expressed lipid transfer protein participating in lipoprotein metabolism in the plasma and tissues [23]. PLTP activity is a risk factor for coronary artery disease [24] and PLTP also plays a role in inflammation and oxidative stress [25].

Taken together, this study demonstrated that the altered expression of ACADM, FABP3, ECH1, ACAA2, EHHADH, CPT1A and PLTP of the PPAR signaling pathway in the left atria of MR patients compared to patients with aortic valve disease and normal controls should play a substantially role in the altered fatty acid metabolism (Fig. 3), glucose metabolism, energy utilization, and pathologic remodeling (hypertrophy, myolysis, glycogen accumulation, apoptosis, autophagy and inflammation) in the atria of MR patients, either partly through altered mitochondrial function, reactive oxidative stress overproduction, inflammation and apoptosis or partly as an adaptive response to volume overload of MR [3, 4, 12, 13, 26].

### Study limitations

There are several limitations in this study. Firstly, the number of study subjects was relatively small. However, the quantitative real-time RT-PCR results were significant and consistent with PCR assay. Secondly, the functional roles of ACADM, FABP3, ECH1, ACAA2, EHHADH, CPT1A and PLTP of the PPAR signaling pathway on the regulation of atrial structural remodeling of MR patients were not specifically examined in this study.

## Conclusions

Differentially expressed genes of the PPAR signaling pathway have been identified in the left atria of MR patients compared to patients with aortic valve disease and normal controls. Moreover, lipid accumulation in the atrial myocytes was significantly increased in the MR patients compared to normal controls. As the PPAR transcriptional regulatory complex controls the expression of fatty acid utilization genes in the myocardium, the results of this study provide rationale for metabolic therapy to remedy atrial structural remodeling associated with atrial enlargement and progression of heart failure in patients with MR.
